# *trans*-Bis(4,7-diphenyl-1,10-phenanthroline-κ^2^ *N*,*N*′)bis­(nitrato-κ^2^ *O*,*O*′)zinc(II)

**DOI:** 10.1107/S1600536810047161

**Published:** 2010-11-20

**Authors:** José A. Fernandes, Filipe A. Almeida Paz, Feng-Yi Liu, Luís Cunha-Silva, Luís D. Carlos, João Rocha

**Affiliations:** a Department of Chemistry, University of Aveiro, CICECO, 3810-193 Aveiro, Portugal; b Department of Physics, University of Aveiro, CICECO, 3810-193 Aveiro, Portugal

## Abstract

The title compound, [Zn(NO_3_)_2_(C_24_H_16_N_2_)_2_], is a twofold axially symmetric coordination compound. Given that the Zn—O interactions [2.4926 (15) and 2.6673 (15) Å] can be considered as weakly bonding and the nitrate ions share the same *C*_2_ axis of the Zn(dpp)_2_ fragment (dpp is 4,7-diphenyl-1,10-phenanthroline), these anions belong to the coordination sphere of Zn^2+^, leading to a complex with an overall coordination number of 8 for the metal ion.

## Related literature

For an isotypic compound containing copper(II), see: Moreno *et al.* (2006 ▶). For structures with eight-coordinate Zn^2+^ ions containing crown ethers, see: Nurtaeva & Holt (2002 ▶); Doxsee *et al.* (1994 ▶); Junk *et al.* (2001 ▶). For structures with eight-coordinate Zn^2+^ ions containing a calyxarene, see: Beer *et al.* (1995 ▶). For structures with eight-coordinate Zn^2+^ ions containing nidoboranes, see: Greenwood *et al.* (1971 ▶); Allmann *et al.* (1976 ▶). For compounds containing the tetra­nitratozincate(II) anion, see: Bellito *et al.* (1976 ▶); Chekhlov (2007 ▶). For a description of the Cambridge Structural Database, see: Allen (2002 ▶). For geometrical aspects of C—H⋯π contacts, see: Babu (2003 ▶). For background research from our group focused on the use of hydro­thermal synthesis to prepare metastable hybrid compounds, see: Paz & Klinowski (2003 ▶, 2004 ▶, 2007 ▶); Paz *et al.* (2005 ▶).
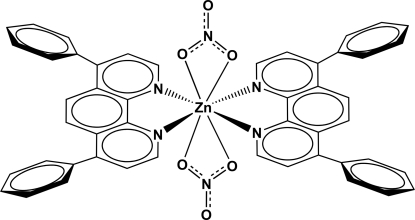


## Experimental

### 

#### Crystal data

[Zn(NO_3_)_2_(C_24_H_16_N_2_)_2_]
*M* *_r_* = 854.17
Monoclinic, 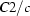

*a* = 20.5074 (4) Å
*b* = 17.4116 (3) Å
*c* = 12.7089 (3) Å
β = 124.035 (1)°
*V* = 3760.56 (13) Å^3^
*Z* = 4
Mo *K*α radiation
μ = 0.72 mm^−1^
*T* = 180 K
0.40 × 0.28 × 0.15 mm

#### Data collection

Bruker X8 Kappa CCD APEXII diffractometer
Absorption correction: multi-scan (*SADABS*; Sheldrick, 1998 ▶) *T*_min_ = 0.762, *T*_max_ = 0.900
36092 measured reflections
4283 independent reflections
3824 reflections with *I* > 2σ(*I*)
*R*_int_ = 0.030

#### Refinement

*R*[*F* ^2^ > 2σ(*F* ^2^)] = 0.029
*wR*(*F* ^2^) = 0.077
*S* = 1.06
4283 reflections
278 parameters
H-atom parameters constrained
Δρ_max_ = 0.29 e Å^−3^
Δρ_min_ = −0.41 e Å^−3^

### 

Data collection: *APEX2* (Bruker, 2006 ▶); cell refinement: *SAINT-Plus* (Bruker, 2005 ▶); data reduction: *SAINT-Plus*; program(s) used to solve structure: *SHELXTL* (Sheldrick, 2008 ▶); program(s) used to refine structure: *SHELXTL*; molecular graphics: *DIAMOND* (Brandenburg, 2009 ▶); software used to prepare material for publication: *SHELXTL*.

## Supplementary Material

Crystal structure: contains datablocks global, I. DOI: 10.1107/S1600536810047161/cv2797sup1.cif

Structure factors: contains datablocks I. DOI: 10.1107/S1600536810047161/cv2797Isup2.hkl

Additional supplementary materials:  crystallographic information; 3D view; checkCIF report

## Figures and Tables

**Fig. 1.**
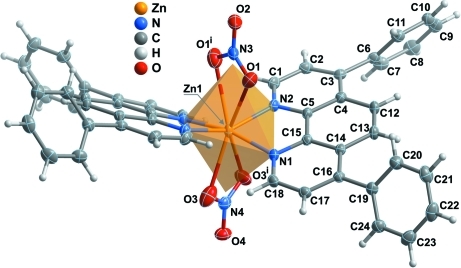
Schematic representation of the molecular unit of the title compound. All non-hydrogen atoms are represented as displacement ellipsoids drawn at the 50% probability level and hydrogen atoms as small spheres with arbitrary radius. Labels are provided for all non-hydrogen atoms composing the asymmetric unit. Symmetry transformation used to generate equivalent atoms is (i) = (-x, y, 0.5 - z).

**Fig. 2.**
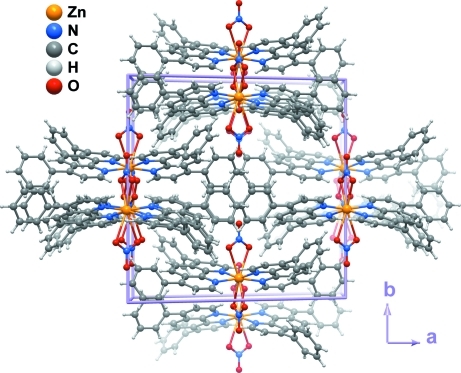
Crystal packing of the title compound viewed in perspective along the [001] direction of the unit cell. Supramolecular interactions (see main text) have been omitted for clarity purposes.

## supplementary crystallographic information

### Comment

Some features of the zinc element, namely small radius and a fully occupied electron *d*-layer make this element unsuitable for preparing compounds with large coordination numbers. A survey in the Cambridge Structural Database (Allen, 2002) revealed, however, some zinc compounds which could be classified as having a coordination number of 8. Nevertheless, most of these compounds comprise loosely bonded cyclic ligands such as crown ethers (Nurtaeva & Holt, 2002; Doxsee *et al.*, 1994; Junk *et al.*, 2001), calyxarenes (Beer *et al.*, 1995) or nidoboranes (Greenwood *et al.*, 1971; Allmann *et al.*, 1976), and also the tetranitratozincate(II) anion (Bellito *et al.*, 1976; Chekhlov *et al.*, 2007). Knowing that the eight-coordinated compound [Cu(dpp)_2_(NO_3_)_2_] (dpp = 4,7-diphenyl-1,10-phenanthroline, C_24_H_16_N_2_) has serendipitously been prepared (Moreno *et al.*, 2006), we decided to test the preparation of the zinc(II) analogue using for that purpose hydrothermal synthetic approaches which have been used systematically in our research group (Paz & Klinowski, 2003; Paz & Klinowski, 2004; Paz & Klinowski, 2007; Paz *et al.*, 2005).

The title compound comprises a twofold axial symmetric Zn^2+^ coordination compound containing two dpp ligands and two nitrato ions (see Scheme). The asymmetric unit comprises half of the complex, in which the metal centre and the O2, N3, N4 and O4 atoms of the nitrato ligands are located in special positions along the rotation axis (Figure 1). The coordination environment around the metal centre can be envisaged as a highly distorted octahedron, where the dpp ligands occupy the equatorial positions and the nitrato ligands are in apical positions. While the Zn—N distances are 2.0843 (12) and 2.1309 (12) Å, the Zn—O ones are instead 2.4926 (15) and 2.6673 (15) Å. The latter values correspond to long Zn—O distances, but nevertheless they are considerably shorter than the Cu—O analogues observed in the isostructural Cu^2+^ compound (Moreno *et al.*, 2006). This feature, in addition to the existence of a common twofold axis with the [Zn(dpp)_2_]^2+^ fragment, is a clear indication that the nitrate is effectively interacting with the metallic centre. The octahedral *cis* and *trans* angles are highly deviated from the ideal value. Considering the centre of gravity (*C_g_*) of the O—N—O chelating moieties as the points where angles are measured, the *cis* angles range from *ca* 76.1 (N2—Zn1···*C_g_*) to 104.89 (4)° (N2—Zn1—N1^i^; symmetry code (i): *-x*, *y*, *0.5 - z*). Conversely, the *trans* angles can be as small as 152.24 (7)° (N2—Zn1—N2^i^). The average planes of the peripheral phenyl moieties of dpp form angles of *ca* 43.4 and 49.6° with the average plane of the phenanthroline fragment.

The crystal structure is rich in weak supramolecular interactions such as π–π stacking, C—H···π (Babu, 2003) and C—H···O. Due to the complexity of the network created by these intermolecular interactions they have been omitted from Figure 2 (crystal packing) for simplicity. Weak π-π stacking interactions occur between pairs of symmetry-equivalent peripheral phenyl substituents, with distances between the centroids (*C_t_*) of *ca* 3.80 and 4.18 Å. C—H/π interactions occur between five H atoms and neighbouring aromatic rings, with 〈(C—H···*C_t_*) larger than *ca* 147° and *d*_H···_*C_t_* in the *ca* 2.80–3.45 Å range. C—H···O hydrogen bonding interactions occur between four H-atoms and neighbouring O-atoms from the nitrato groups: 〈(DHA) larger than *ca* 140° and internuclear D···A distances in the *ca* 3.29–3.50 Å range.

### Experimental

Starting chemicals were purchased from commercial sources and were used as received without any further purification. The title compound was prepared in a Teflon-lined reaction vessel under static hydrothermal conditions in an oven preheated at 160 °C. The total reaction time was of 2 days. The reactive mixture was prepared by using a Zn(NO_3_)_2_.6H_2_O: dpp (4,7-diphenyl-1,10-phenanthroline) molar ratio of about 2: 1. After reacting, a large amount of brown-red crystals could be directly isolated from the contents of the reaction vessel.

### Refinement

Hydrogen atoms bound to aromatic carbon atoms were located at their idealized positions and were included in the final structural model in riding-motion approximation with C—H = 0.95 Å. The isotropic displacement parameters for these atoms were fixed at 1.2 times *U*_eq_ of the respective parent carbon atom.

### Crystal data

**Table:**

[Zn(NO_3_)_2_(C_24_H_16_N_2_)_2_]	*F*(000) = 1760
*M**_r_* = 854.17	*D*_x_ = 1.509 Mg m^−^^3^
Monoclinic, *C*2/*c*	Mo *K*α radiation, λ = 0.71073 Å
Hall symbol: -C 2yc	Cell parameters from 9894 reflections
*a* = 20.5074 (4) Å	θ = 3.0–30.2°
*b* = 17.4116 (3) Å	µ = 0.72 mm^−^^1^
*c* = 12.7089 (3) Å	*T* = 180 K
β = 124.035 (1)°	Prism, colourless
*V* = 3760.56 (13) Å^3^	0.40 × 0.28 × 0.15 mm
*Z* = 4	

### Data collection

**Table:**

Bruker X8 Kappa CCD APEXII diffractometer	4283 independent reflections
Radiation source: fine-focus sealed tube	3824 reflections with *I* > 2σ(*I*)
graphite	*R*_int_ = 0.030
ω and φ scans	θ_max_ = 27.5°, θ_min_ = 3.6°
Absorption correction: multi-scan (*SADABS*; Sheldrick, 1998)	*h* = −26→26
*T*_min_ = 0.762, *T*_max_ = 0.900	*k* = −22→20
36092 measured reflections	*l* = −16→16

### Refinement

**Table:**

Refinement on *F*^2^	Primary atom site location: structure-invariant direct methods
Least-squares matrix: full	Secondary atom site location: difference Fourier map
*R*[*F*^2^ > 2σ(*F*^2^)] = 0.029	Hydrogen site location: inferred from neighbouring sites
*wR*(*F*^2^) = 0.077	H-atom parameters constrained
*S* = 1.06	*w* = 1/[σ^2^(*F*_o_^2^) + (0.0361*P*)^2^ + 3.6556*P*] where *P* = (*F*_o_^2^ + 2*F*_c_^2^)/3
4283 reflections	(Δ/σ)_max_ = 0.001
278 parameters	Δρ_max_ = 0.29 e Å^−^^3^
0 restraints	Δρ_min_ = −0.41 e Å^−^^3^

### Special details

**Table:**

Geometry. All e.s.d.'s (except the e.s.d. in the dihedral angle between two l.s. planes) are estimated using the full covariance matrix. The cell e.s.d.'s are taken into account individually in the estimation of e.s.d.'s in distances, angles and torsion angles; correlations between e.s.d.'s in cell parameters are only used when they are defined by crystal symmetry. An approximate (isotropic) treatment of cell e.s.d.'s is used for estimating e.s.d.'s involving l.s. planes.
Refinement. Refinement of *F*^2^ against ALL reflections. The weighted *R*-factor *wR* and goodness of fit *S* are based on *F*^2^, conventional *R*-factors *R* are based on *F*, with *F* set to zero for negative *F*^2^. The threshold expression of *F*^2^ > σ(*F*^2^) is used only for calculating *R*-factors(gt) *etc*. and is not relevant to the choice of reflections for refinement. *R*-factors based on *F*^2^ are statistically about twice as large as those based on *F*, and *R*- factors based on ALL data will be even larger.

### Fractional atomic coordinates and isotropic or equivalent isotropic displacement parameters (Å^2^)

**Table:**

	*x*	*y*	*z*	*U*_iso_*/*U*_eq_	
Zn1	0.0000	0.406835 (14)	0.2500	0.02344 (8)	
N1	0.10982 (7)	0.42246 (7)	0.27115 (11)	0.0191 (2)	
N2	0.07903 (7)	0.37812 (7)	0.44046 (11)	0.0205 (2)	
C1	0.06298 (8)	0.36151 (8)	0.52558 (15)	0.0239 (3)	
H1	0.0096	0.3623	0.4996	0.029*	
C2	0.12052 (9)	0.34307 (8)	0.65081 (14)	0.0236 (3)	
H2	0.1063	0.3345	0.7093	0.028*	
C3	0.19831 (8)	0.33704 (8)	0.69094 (13)	0.0190 (3)	
C4	0.21717 (8)	0.35236 (8)	0.60058 (13)	0.0175 (3)	
C5	0.15537 (8)	0.37498 (8)	0.47776 (13)	0.0173 (3)	
C6	0.25883 (8)	0.31729 (8)	0.82531 (13)	0.0205 (3)	
C7	0.26247 (9)	0.35967 (9)	0.92152 (15)	0.0269 (3)	
H7	0.2273	0.4014	0.9005	0.032*	
C8	0.31732 (10)	0.34113 (10)	1.04812 (15)	0.0316 (4)	
H8	0.3204	0.3709	1.1134	0.038*	
C9	0.36722 (9)	0.27957 (10)	1.07902 (14)	0.0295 (3)	
H9	0.4045	0.2669	1.1656	0.035*	
C10	0.36317 (9)	0.23612 (9)	0.98444 (15)	0.0269 (3)	
H10	0.3968	0.1930	1.0061	0.032*	
C11	0.30981 (8)	0.25548 (9)	0.85766 (14)	0.0233 (3)	
H11	0.3081	0.2264	0.7929	0.028*	
C12	0.29576 (8)	0.35127 (8)	0.63016 (13)	0.0194 (3)	
H12	0.3378	0.3344	0.7117	0.023*	
C13	0.31121 (8)	0.37373 (8)	0.54435 (13)	0.0199 (3)	
H13	0.3641	0.3735	0.5679	0.024*	
C14	0.24988 (8)	0.39783 (8)	0.41882 (13)	0.0174 (3)	
C15	0.17192 (8)	0.39831 (7)	0.38575 (13)	0.0173 (3)	
C16	0.26372 (8)	0.42396 (8)	0.32660 (13)	0.0180 (3)	
C17	0.19943 (8)	0.45022 (8)	0.21198 (13)	0.0212 (3)	
H17	0.2066	0.4695	0.1492	0.025*	
C18	0.12428 (8)	0.44864 (8)	0.18798 (13)	0.0218 (3)	
H18	0.0814	0.4671	0.1085	0.026*	
C19	0.34289 (8)	0.42377 (8)	0.34930 (13)	0.0200 (3)	
C20	0.39130 (9)	0.35907 (9)	0.39623 (14)	0.0248 (3)	
H20	0.3747	0.3145	0.4182	0.030*	
C21	0.46359 (9)	0.35970 (10)	0.41090 (15)	0.0317 (4)	
H21	0.4963	0.3155	0.4429	0.038*	
C22	0.48832 (9)	0.42424 (11)	0.37928 (16)	0.0337 (4)	
H22	0.5378	0.4242	0.3893	0.040*	
C23	0.44103 (10)	0.48887 (10)	0.33299 (16)	0.0323 (4)	
H23	0.4580	0.5333	0.3115	0.039*	
C24	0.36867 (9)	0.48850 (9)	0.31812 (14)	0.0260 (3)	
H24	0.3363	0.5329	0.2863	0.031*	
N3	0.0000	0.22985 (10)	0.2500	0.0302 (4)	
O1	0.04653 (7)	0.26663 (8)	0.23472 (13)	0.0460 (3)	
O2	0.0000	0.15898 (9)	0.2500	0.0450 (5)	
N4	0.0000	0.57342 (10)	0.2500	0.0246 (4)	
O3	−0.02609 (8)	0.53615 (8)	0.14987 (13)	0.0462 (3)	
O4	0.0000	0.64379 (9)	0.2500	0.0333 (4)	

### Atomic displacement parameters (Å^2^)

**Table:**

	*U*^11^	*U*^22^	*U*^33^	*U*^12^	*U*^13^	*U*^23^
Zn1	0.01213 (12)	0.03237 (15)	0.02007 (13)	0.000	0.00548 (10)	0.000
N1	0.0154 (5)	0.0210 (6)	0.0167 (6)	−0.0005 (4)	0.0065 (5)	0.0001 (4)
N2	0.0158 (5)	0.0210 (6)	0.0245 (6)	0.0004 (4)	0.0112 (5)	0.0015 (5)
C1	0.0180 (7)	0.0255 (7)	0.0323 (8)	0.0018 (5)	0.0166 (6)	0.0045 (6)
C2	0.0257 (7)	0.0235 (7)	0.0294 (8)	0.0018 (6)	0.0203 (7)	0.0041 (6)
C3	0.0216 (7)	0.0164 (6)	0.0209 (7)	−0.0008 (5)	0.0131 (6)	−0.0004 (5)
C4	0.0172 (6)	0.0177 (6)	0.0182 (6)	−0.0007 (5)	0.0104 (5)	−0.0018 (5)
C5	0.0149 (6)	0.0170 (6)	0.0193 (6)	−0.0009 (5)	0.0092 (5)	−0.0015 (5)
C6	0.0215 (7)	0.0235 (7)	0.0201 (7)	−0.0034 (5)	0.0140 (6)	0.0012 (5)
C7	0.0327 (8)	0.0280 (8)	0.0268 (8)	−0.0003 (6)	0.0208 (7)	−0.0004 (6)
C8	0.0410 (9)	0.0375 (9)	0.0227 (8)	−0.0079 (7)	0.0218 (7)	−0.0054 (6)
C9	0.0281 (8)	0.0394 (9)	0.0190 (7)	−0.0069 (7)	0.0120 (6)	0.0045 (6)
C10	0.0241 (7)	0.0301 (8)	0.0264 (8)	0.0008 (6)	0.0140 (6)	0.0061 (6)
C11	0.0248 (7)	0.0250 (7)	0.0223 (7)	−0.0009 (6)	0.0144 (6)	0.0007 (6)
C12	0.0148 (6)	0.0247 (7)	0.0154 (6)	0.0015 (5)	0.0064 (5)	0.0003 (5)
C13	0.0137 (6)	0.0263 (7)	0.0187 (7)	0.0011 (5)	0.0084 (6)	−0.0003 (5)
C14	0.0165 (6)	0.0187 (6)	0.0165 (6)	−0.0005 (5)	0.0089 (5)	−0.0016 (5)
C15	0.0159 (6)	0.0166 (6)	0.0171 (6)	−0.0009 (5)	0.0079 (5)	−0.0015 (5)
C16	0.0193 (7)	0.0168 (6)	0.0188 (6)	−0.0007 (5)	0.0112 (6)	−0.0014 (5)
C17	0.0234 (7)	0.0221 (7)	0.0188 (7)	−0.0001 (5)	0.0123 (6)	0.0022 (5)
C18	0.0203 (7)	0.0225 (7)	0.0163 (6)	0.0007 (5)	0.0064 (6)	0.0021 (5)
C19	0.0199 (7)	0.0252 (7)	0.0168 (6)	−0.0003 (5)	0.0115 (6)	−0.0013 (5)
C20	0.0268 (8)	0.0258 (7)	0.0259 (7)	0.0030 (6)	0.0174 (6)	0.0010 (6)
C21	0.0269 (8)	0.0404 (9)	0.0306 (8)	0.0102 (7)	0.0179 (7)	0.0019 (7)
C22	0.0219 (8)	0.0546 (11)	0.0298 (8)	−0.0020 (7)	0.0178 (7)	−0.0050 (7)
C23	0.0311 (9)	0.0404 (9)	0.0322 (8)	−0.0079 (7)	0.0219 (7)	−0.0007 (7)
C24	0.0271 (8)	0.0274 (8)	0.0265 (8)	0.0000 (6)	0.0169 (7)	0.0026 (6)
N3	0.0194 (9)	0.0234 (9)	0.0284 (10)	0.000	0.0016 (8)	0.000
O1	0.0320 (7)	0.0442 (8)	0.0517 (8)	−0.0079 (6)	0.0172 (6)	0.0062 (6)
O2	0.0322 (9)	0.0204 (8)	0.0539 (11)	0.000	0.0066 (8)	0.000
N4	0.0198 (8)	0.0247 (9)	0.0339 (10)	0.000	0.0178 (8)	0.000
O3	0.0391 (7)	0.0525 (8)	0.0517 (8)	−0.0126 (6)	0.0283 (7)	−0.0281 (7)
O4	0.0374 (9)	0.0207 (8)	0.0483 (10)	0.000	0.0278 (8)	0.000

### Geometric parameters (Å, °)

**Table:**

Zn1—N2^i^	2.0843 (12)	C10—H10	0.9500
Zn1—N2	2.0843 (12)	C11—H11	0.9500
Zn1—N1	2.1309 (12)	C12—C13	1.352 (2)
Zn1—N1^i^	2.1309 (12)	C12—H12	0.9500
Zn1—O3^i^	2.4926 (15)	C13—C14	1.4343 (19)
Zn1—O3	2.4926 (15)	C13—H13	0.9500
Zn1—O1^i^	2.6673 (15)	C14—C15	1.4073 (19)
Zn1—O1	2.6673 (15)	C14—C16	1.4261 (19)
N1—C18	1.3282 (18)	C16—C17	1.3854 (19)
N1—C15	1.3578 (17)	C16—C19	1.4814 (19)
N2—C1	1.3270 (19)	C17—C18	1.394 (2)
N2—C5	1.3594 (17)	C17—H17	0.9500
C1—C2	1.388 (2)	C18—H18	0.9500
C1—H1	0.9500	C19—C24	1.393 (2)
C2—C3	1.3803 (19)	C19—C20	1.396 (2)
C2—H2	0.9500	C20—C21	1.387 (2)
C3—C4	1.4271 (19)	C20—H20	0.9500
C3—C6	1.4840 (19)	C21—C22	1.382 (3)
C4—C5	1.4083 (19)	C21—H21	0.9500
C4—C12	1.4356 (18)	C22—C23	1.383 (3)
C5—C15	1.4464 (19)	C22—H22	0.9500
C6—C11	1.392 (2)	C23—C24	1.387 (2)
C6—C7	1.394 (2)	C23—H23	0.9500
C7—C8	1.390 (2)	C24—H24	0.9500
C7—H7	0.9500	N3—O2	1.234 (2)
C8—C9	1.379 (2)	N3—O1	1.2513 (16)
C8—H8	0.9500	N3—O1^i^	1.2513 (16)
C9—C10	1.383 (2)	N4—O4	1.225 (2)
C9—H9	0.9500	N4—O3	1.2508 (15)
C10—C11	1.390 (2)	N4—O3^i^	1.2508 (15)
			
N2^i^—Zn1—N2	152.24 (7)	C7—C8—H8	120.0
N2^i^—Zn1—N1	104.89 (4)	C8—C9—C10	120.26 (14)
N2—Zn1—N1	78.71 (4)	C8—C9—H9	119.9
N2^i^—Zn1—N1^i^	78.71 (4)	C10—C9—H9	119.9
N2—Zn1—N1^i^	104.89 (4)	C9—C10—C11	120.00 (15)
N1—Zn1—N1^i^	165.33 (6)	C9—C10—H10	120.0
N2^i^—Zn1—O3^i^	127.93 (4)	C11—C10—H10	120.0
N2—Zn1—O3^i^	79.55 (4)	C10—C11—C6	120.23 (14)
N1—Zn1—O3^i^	84.87 (4)	C10—C11—H11	119.9
N1^i^—Zn1—O3^i^	81.88 (4)	C6—C11—H11	119.9
N2^i^—Zn1—O3	79.55 (4)	C13—C12—C4	121.36 (12)
N2—Zn1—O3	127.93 (4)	C13—C12—H12	119.3
N1—Zn1—O3	81.88 (4)	C4—C12—H12	119.3
N1^i^—Zn1—O3	84.87 (4)	C12—C13—C14	121.75 (12)
O3^i^—Zn1—O3	50.81 (6)	C12—C13—H13	119.1
N2^i^—Zn1—O1^i^	77.74 (5)	C14—C13—H13	119.1
N2—Zn1—O1^i^	76.89 (4)	C15—C14—C16	117.91 (12)
N1—Zn1—O1^i^	120.24 (4)	C15—C14—C13	118.44 (12)
N1^i^—Zn1—O1^i^	74.34 (4)	C16—C14—C13	123.61 (12)
O3^i^—Zn1—O1^i^	140.61 (4)	N1—C15—C14	123.29 (12)
O3—Zn1—O1^i^	151.71 (4)	N1—C15—C5	116.78 (12)
N2^i^—Zn1—O1	76.89 (4)	C14—C15—C5	119.89 (12)
N2—Zn1—O1	77.74 (5)	C17—C16—C14	117.34 (12)
N1—Zn1—O1	74.34 (4)	C17—C16—C19	119.99 (12)
N1^i^—Zn1—O1	120.24 (4)	C14—C16—C19	122.67 (12)
O3^i^—Zn1—O1	151.71 (4)	C16—C17—C18	120.58 (13)
O3—Zn1—O1	140.61 (4)	C16—C17—H17	119.7
O1^i^—Zn1—O1	47.53 (6)	C18—C17—H17	119.7
C18—N1—C15	117.83 (12)	N1—C18—C17	123.00 (13)
C18—N1—Zn1	129.38 (9)	N1—C18—H18	118.5
C15—N1—Zn1	112.73 (9)	C17—C18—H18	118.5
C1—N2—C5	118.01 (12)	C24—C19—C20	118.83 (13)
C1—N2—Zn1	127.78 (10)	C24—C19—C16	119.45 (13)
C5—N2—Zn1	114.19 (9)	C20—C19—C16	121.66 (13)
N2—C1—C2	123.07 (13)	C21—C20—C19	120.15 (14)
N2—C1—H1	118.5	C21—C20—H20	119.9
C2—C1—H1	118.5	C19—C20—H20	119.9
C3—C2—C1	120.41 (13)	C22—C21—C20	120.40 (15)
C3—C2—H2	119.8	C22—C21—H21	119.8
C1—C2—H2	119.8	C20—C21—H21	119.8
C2—C3—C4	117.79 (13)	C21—C22—C23	120.07 (15)
C2—C3—C6	119.47 (12)	C21—C22—H22	120.0
C4—C3—C6	122.72 (12)	C23—C22—H22	120.0
C5—C4—C3	117.58 (12)	C22—C23—C24	119.74 (15)
C5—C4—C12	118.42 (12)	C22—C23—H23	120.1
C3—C4—C12	123.85 (12)	C24—C23—H23	120.1
N2—C5—C4	122.97 (12)	C23—C24—C19	120.81 (15)
N2—C5—C15	116.89 (12)	C23—C24—H24	119.6
C4—C5—C15	120.12 (12)	C19—C24—H24	119.6
C11—C6—C7	119.16 (13)	O2—N3—O1	120.79 (10)
C11—C6—C3	121.67 (13)	O2—N3—O1^i^	120.79 (10)
C7—C6—C3	119.11 (13)	O1—N3—O1^i^	118.4 (2)
C8—C7—C6	120.25 (15)	N3—O1—Zn1	97.02 (11)
C8—C7—H7	119.9	O4—N4—O3	121.25 (10)
C6—C7—H7	119.9	O4—N4—O3^i^	121.25 (10)
C9—C8—C7	120.07 (15)	O3—N4—O3^i^	117.5 (2)
C9—C8—H8	120.0	N4—O3—Zn1	95.84 (11)
			
N2^i^—Zn1—N1—C18	32.48 (13)	C5—C4—C12—C13	2.2 (2)
N2—Zn1—N1—C18	−175.80 (13)	C3—C4—C12—C13	−173.24 (13)
N1^i^—Zn1—N1—C18	−70.00 (12)	C4—C12—C13—C14	−1.6 (2)
O3^i^—Zn1—N1—C18	−95.46 (12)	C12—C13—C14—C15	0.4 (2)
O3—Zn1—N1—C18	−44.37 (12)	C12—C13—C14—C16	178.20 (13)
O1^i^—Zn1—N1—C18	116.78 (12)	C18—N1—C15—C14	−1.7 (2)
O1—Zn1—N1—C18	103.92 (13)	Zn1—N1—C15—C14	175.54 (10)
N2^i^—Zn1—N1—C15	−144.42 (9)	C18—N1—C15—C5	175.91 (12)
N2—Zn1—N1—C15	7.31 (9)	Zn1—N1—C15—C5	−6.80 (15)
N1^i^—Zn1—N1—C15	113.11 (9)	C16—C14—C15—N1	−0.2 (2)
O3^i^—Zn1—N1—C15	87.65 (9)	C13—C14—C15—N1	177.73 (12)
O3—Zn1—N1—C15	138.74 (10)	C16—C14—C15—C5	−177.75 (12)
O1^i^—Zn1—N1—C15	−60.11 (10)	C13—C14—C15—C5	0.14 (19)
O1—Zn1—N1—C15	−72.97 (9)	N2—C5—C15—N1	1.08 (18)
N2^i^—Zn1—N2—C1	−84.68 (12)	C4—C5—C15—N1	−177.28 (12)
N1—Zn1—N2—C1	174.74 (13)	N2—C5—C15—C14	178.82 (12)
N1^i^—Zn1—N2—C1	9.35 (14)	C4—C5—C15—C14	0.46 (19)
O3^i^—Zn1—N2—C1	87.95 (13)	C15—C14—C16—C17	1.90 (19)
O3—Zn1—N2—C1	104.51 (13)	C13—C14—C16—C17	−175.87 (13)
O1^i^—Zn1—N2—C1	−60.25 (12)	C15—C14—C16—C19	−178.05 (12)
O1—Zn1—N2—C1	−109.04 (13)	C13—C14—C16—C19	4.2 (2)
N2^i^—Zn1—N2—C5	93.76 (9)	C14—C16—C17—C18	−1.8 (2)
N1—Zn1—N2—C5	−6.81 (9)	C19—C16—C17—C18	178.14 (13)
N1^i^—Zn1—N2—C5	−172.20 (9)	C15—N1—C18—C17	1.9 (2)
O3^i^—Zn1—N2—C5	−93.60 (10)	Zn1—N1—C18—C17	−174.87 (10)
O3—Zn1—N2—C5	−77.04 (11)	C16—C17—C18—N1	−0.1 (2)
O1^i^—Zn1—N2—C5	118.20 (10)	C17—C16—C19—C24	46.16 (19)
O1—Zn1—N2—C5	69.41 (10)	C14—C16—C19—C24	−133.88 (14)
C5—N2—C1—C2	1.6 (2)	C17—C16—C19—C20	−131.04 (15)
Zn1—N2—C1—C2	−179.98 (11)	C14—C16—C19—C20	48.91 (19)
N2—C1—C2—C3	−3.7 (2)	C24—C19—C20—C21	−0.2 (2)
C1—C2—C3—C4	1.6 (2)	C16—C19—C20—C21	177.05 (13)
C1—C2—C3—C6	179.98 (13)	C19—C20—C21—C22	0.0 (2)
C2—C3—C4—C5	2.00 (19)	C20—C21—C22—C23	0.2 (3)
C6—C3—C4—C5	−176.28 (12)	C21—C22—C23—C24	−0.2 (3)
C2—C3—C4—C12	177.44 (13)	C22—C23—C24—C19	0.0 (2)
C6—C3—C4—C12	−0.8 (2)	C20—C19—C24—C23	0.2 (2)
C1—N2—C5—C4	2.3 (2)	C16—C19—C24—C23	−177.13 (14)
Zn1—N2—C5—C4	−176.27 (10)	O2—N3—O1—Zn1	180.0
C1—N2—C5—C15	−175.98 (12)	O1^i^—N3—O1—Zn1	0.0
Zn1—N2—C5—C15	5.42 (15)	N2^i^—Zn1—O1—N3	−85.27 (7)
C3—C4—C5—N2	−4.1 (2)	N2—Zn1—O1—N3	83.35 (8)
C12—C4—C5—N2	−179.82 (12)	N1—Zn1—O1—N3	164.89 (8)
C3—C4—C5—C15	174.13 (12)	N1^i^—Zn1—O1—N3	−16.89 (9)
C12—C4—C5—C15	−1.56 (19)	O3^i^—Zn1—O1—N3	120.67 (8)
C2—C3—C6—C11	125.56 (15)	O3—Zn1—O1—N3	−140.03 (7)
C4—C3—C6—C11	−56.18 (19)	O1^i^—Zn1—O1—N3	0.0
C2—C3—C6—C7	−51.64 (19)	O4—N4—O3—Zn1	180.0
C4—C3—C6—C7	126.62 (15)	O3^i^—N4—O3—Zn1	0.0
C11—C6—C7—C8	1.1 (2)	N2^i^—Zn1—O3—N4	163.13 (7)
C3—C6—C7—C8	178.40 (13)	N2—Zn1—O3—N4	−21.21 (9)
C6—C7—C8—C9	−1.5 (2)	N1—Zn1—O3—N4	−89.99 (7)
C7—C8—C9—C10	0.2 (2)	N1^i^—Zn1—O3—N4	83.70 (7)
C8—C9—C10—C11	1.4 (2)	O3^i^—Zn1—O3—N4	0.0
C9—C10—C11—C6	−1.8 (2)	O1^i^—Zn1—O3—N4	126.09 (8)
C7—C6—C11—C10	0.5 (2)	O1—Zn1—O3—N4	−142.88 (6)
C3—C6—C11—C10	−176.71 (13)		

Symmetry codes: (i) −*x*, *y*, −*z*+1/2.
